# Whole Genome Comparison of *Thermus* sp. NMX2.A1 Reveals Principal Carbon Metabolism Differences with Closest Relation *Thermus scotoductus* SA-01

**DOI:** 10.1534/g3.116.032953

**Published:** 2016-07-11

**Authors:** Walter J. Müller, Nokuthula Tlalajoe, Errol D. Cason, Derek Litthauer, Oleg Reva, Elzbieta Brzuszkiewicz, Esta van Heerden

**Affiliations:** * Department of Microbial, Biochemical and Food Biotechnology, University of the Free State, Bloemfontein 9301, South Africa; † National Control Laboratory for Biological Products, University of the Free State, Bloemfontein 9301, South Africa; ‡ Centre for Bioinformatics and Computational Biology, Department of Biochemistry, University of Pretoria, Hatfield 0028, South Africa; § Department of Genomic and Applied Microbiology, Georg-August-Universität Göttingen, 37073, Germany

**Keywords:** *Thermus* sp. NMX2.A1, *Thermus scotoductus* SA-01, genome, Roche 454, comparison, Calvin–Benson–Bassham

## Abstract

Genome sequencing of the yellow-pigmented, thermophilic bacterium *Thermus* sp. NMX2.A1 resulted in a 2.29 Mb draft genome that encodes for 2312 proteins. The genetic relationship between various strains from the genus *Thermus* was assessed based on phylogenomic analyses using a concatenated set of conserved proteins. The resulting phylogenetic tree illustrated that *Thermus* sp. NMX2 A.1 clusters together with *Thermus scotoductus* SA-01, despite being isolated from vastly different geographical locations. The close evolutionary relationship and metabolic parallels between the two strains has previously been recognized; however, neither strain’s genome data were available at that point in time. Genomic comparison of the *Thermus* sp. NMX2.A1 and *T. scotoductus* SA-01, as well as other closely related *Thermus* strains, revealed a high degree of synteny at both the genomic and proteomic level, with processes such as denitrification and natural cell competence appearing to be conserved. However, despite this high level of similarity, analysis revealed a complete, putative Calvin–Benson–Bassham (CBB) cycle in NMX2.A1 that is absent in SA-01. Analysis of horizontally transferred gene islands provide evidence that NMX2 selected these genes due to pressure from its HCO_3_^-^ rich environment, which is in stark contrast to that of the deep subsurface isolated SA-01.

The rapid growth in the number of completely sequenced bacterial and archaeal genomes has made the field of comparative genomics more feasible than ever. This, in turn, has started to highlight the importance of studying the microevolutionary relationships between closely related genomes. Microevolutionary processes are central to our understanding of the mechanisms of evolution, specifically the differential effects of selection on different types of genomic sequences (Jordan *et al.* 2002). 

Databases contain multiple sets of closely related genomes from the genus *Thermus* for microevolutionary studies (Chung *et al.* 2000; Bricio *et al.* 2011). Especially relevant to this study are the strains *Thermus* sp. NMX2. A.1 and *T. scotoductus* SA-01. These two strains, isolated from a hot spring in New Mexico and a deep gold mine in South Africa, respectively, share high 16S rRNA gene sequence similarity (> 98%) in spite of their vast geographical separation (Hudson *et al.* 1989; Kieft *et al.* 1999). Furthermore, Kieft *et al.* (1999) showed that the two strains displayed distinct metabolic similarities: both strains were able to grow on lactate with O_2_, NO_3_^-^, Mn(IV), S^0^, and Fe(III)-NTA as terminal electron acceptors and both grew optimally at ∼65° (pH 6.5–7.0). Dissimilatory reduction of iron (Bester *et al.* 2010) and other metals by unique protein functionalities (Opperman and Van Heerden 2007; Cason *et al.* 2012) in these two *Thermus* strains is unusual within this genus, thus prompting further microevolutionary studies. 

The genome of *T. scotoductus* SA-01 was recently fully sequenced and assembled (Gounder *et al.* 2011). Along with the genome sequence of *Thermus* sp. NMX2 A.1, presented in this paper, an opportunity now exists to identify the genetic differences between these metabolically similar strains, as well as other available *Thermus* genomes. In the present study we illustrate that, despite the genomic similarities of *T. scotoductus* SA-01 and *Thermus* sp. NMX2 A.1, they do harbor unexplored species differences when compared to each other as well as with other members of the genus *Thermus*. The most striking differences were observed when comparing the genes of the putative CBB cycle.

## Materials and Methods

### Genome sequence and annotation

*Thermus* sp. NMX2.A1 was generously provided by Prof. T. L. Kieft (New Mexico Tech) (Hudson *et al.* 1989). Cell mass for DNA isolation was obtained by aerobic culture in ATTC medium 697 (4 g/L yeast extract, 8 g/L peptone, and 2 g/L NaCl; pH 7.5) at 68°. The bacterial strain was verified by 16S rRNA gene sequencing and comparing it to the deposited GenBank sequence (accession number L09661.1). Genomic DNA of *Thermus* sp. NMX2 A.1 was sequenced by GATC Biotech using a 1/4 FLX plate, providing an approximate 87-fold coverage. *De novo* assembly was performed using the Roche 454 Newbler software. Annotation was performed using the NCBI Prokaryotic Genome Annotation Pipeline (released 2013).

### Identification and analysis of horizontally acquired genomic islands

Identification of genomic islands in concatenated contigs was performed using SeqWord Gene Island Sniffer (SWGIS) (Bezuidt *et al.* 2009). Genomic islands sharing DNA sequence similarities were searched by BLASTN through the Pre_GI database (Pierneef *et al.* 2015).

### Phylogenomic inference

Complete genome sequences of *Thermus* sp. NMX2 A.1 strain was compared against complete genomes of 10 reference *Thermus*/*Meiothermus* strains (*T. scotoductus* SA-01, *T. igniterrae* ATTC 700962, *Thermus* sp. CBB US3 UF1, *T. aquaticus* Y51MC23, *Thermus* sp. RL, *T. thermophilus* HB27, *T. thermophilus* HB8, *T. oshimai* JL-2, *M. ruber* DSM 1279, and *M. silvanus* DSM 9946). BLASTP search revealed 1389 clusters of orthologous proteins shared by all these organisms. All protein sequences were aligned by MUSCLE and each alignment was edited by Gblocks (Talavera and Castresana 2007). The resulting edited alignments were concatenated into an artificial amino acid superalignment of 377,241 residues. A phylogenetic tree was inferred by the Neighbor-Joining algorithm implemented in MEGA 6.0 (Tamura *et al.* 2013).

### Homologous protein phylogenetic tree

Three sequential orthologous sequences from the CBB cycle from *Thermus* spp. were manually concatenated into an artificial amino acid sequence. The selected proteins were: ribulose bisphosphate carboxylase small chain (EC 4.1.1.39), ribulose bisphosphate carboxylase large chain (EC 4.1.1.39), and the fructose-1,6-bisphosphate, GlpX type (EC 3.1.3.11). *Thermus* strains utilized were *T. antranikianii* DSM 12462, *T. igniterrae* ATCC 700962, *T. islandicus* DSM 21543, *T. oshimai* DSM 12092, *T. oshimai* JL-2, *Thermus* sp. NMX2.A1, and *Thermus* sp. YIM 77409. A phylogenetic tree was inferred by the Neighbor-Joining algorithm implemented in MEGA 6.0 using *Paracoccus yeei* ATCC BAA-599 as an outlier group (Tamura *et al.* 2013). 

### Chromosomal alignment

Contigs obtained from the *de novo* assembly were aligned and ordered with MAUVE by using the *T. scotoductus* SA-01 chromosome as template (Darling *et al.* 2004).

### Bidirectional BLAST

The annotated proteome from the *Thermus* sp. NMX2 A.1 contigs was compared with those from other *Thermus* strains: *T. scotoductus* SA-01, *T. scotoductus* KI2, *T. scotoductus* DSM 8553, *T. thermophilus* HB-27, *T. thermophilus* HB-8, *T*. *thermophilus* JL-18, *T. thermophiles* ATCC 33923, *T. thermophiles* SGO.5JP17-16, *T. antranikianii* DSM 12462, *T. igniterrae* ATCC 700962, *T. oshimai* DSM 12092, *T. oshimai* JL-2, *Thermus* sp., *Thermus* sp. 2.9, *Thermus* sp. CCB_US3_UF1, *Thermus* sp. RL, YIM 77409, and *T. islandicus* DSM 21543. The bidirectional best hits were selected using the proteome comparison tool in PATRIC (https://www.patricbrc.org) (Wattam *et al.* 2014).

### Pathway Genome Database construction

A Pathway Genome Database (PGDB) was constructed using the Pathologic module of the Pathway Tools software (http://brg.ai.sri.com/ptools/) (Karp 2001; Karp *et al.* 2002). Genes and proteins were imported into Pathologic from the annotated genome sequences in Genbank file format and MetaCyc (Caspi *et al.* 2014) was selected as the reference pathway database. Comparisons of the putative CBB cycles of *Thermus* sp. NMX2.A1 and *Thermus* spp. available in the BioCyc PGDB database (*T. antranikianii* DSM 12462, *T. igniterrae* ATCC 700962, *T. islandicus* DSM 21543, *T. oshimai* JL-2, *T. scotoductus* SA-01, *T. aquaticus* Y51MC23, *T. caliditerrae*, *T. filiformis*, *T*. sp. CCB_US3_UF1, *T. thermophilus* HB27, *T. thermophilus* HB8, *T. thermophilus* JL-18, and *T. thermophilus* SG0.5JP17-16) were performed using the Species Compare tool in Pathway Tools.

### Data availability

The authors state that all data necessary for confirming the conclusions presented in the article are represented fully within the article.

## Results and Discussion

### Assembly of sequenced data

The results for the *de novo* genome assembly yielded 132 contigs and showed an approximate genome size of 2293 Mbp (NCBI accession ATNI00000000.1), which is similar to the genome size of *T. scotoductus* SA-01 consisting of 2346 Mbp. Table 1 illustrates some genome features of sequenced *Thermus* strains including *Thermus* sp. NMX2.A1.

**Table 1:** Comparison of genome features of sequenced *Thermus* strains (taken from NCBI)

*Thermus* Strain	Chromosome Size (Mb)	Plasmid Name	Plasmid Size (bp)	GC %	tRNA	rRNA	Other RNA	Genes	Proteins	Pseudogenes
*T. aquaticus Y51MC23*	2.34			68	50	4		2593	2539	
*T. antranikianii DSM 12462*	2.16			64.8	47	8	1	2298	2220	22
*T. caliditerrae YIM 77777*	2.22			67.2	50	8	1	2306	2198	49
*T. filiformis ATT43280*	2.39			69	47	6	1	2405	2211	140
*T. igniterrae ATCC 700962*	2.23			68.8	43	7	1	2339	2276	12
*T. islandicus DSM 21543*	2.26			68.3	47	9	1	2401	2303	41
*T. oshimai JL-2*	2.07			68.6	50	6	2	2188	2116	14
		pTHEOS01	271,713	68.6				265	262	3
		pTHEOS02	57,223	68.4		1		72	69	2
*Thermus* sp. NMX2.A1	2.29			65.3	43	7	2	2469	2312	105
*T. scotoductus SA-01*	2.35			64.9	48	5	1	2467	2384	29
		pTSC8	8383	65.9				13	12	1
*T. scotoductus KI2*	2.48			65.5	47	1		2611	2418	139
*T. scotoductus DSM 8553*	2.07			64.8	47	8	1	2242	2156	30
*T. tengchongensis YIM 77401 (T. yunnanensis)*	2.56			66.4	47	6	1	2729	2552	123
*T. thermophilus HB27*	1.89			69.4	49	6	1	2027	1939	32
		pTT27	232,605	69.2				224	213	11
*T. thermophilus HB8*	1.85			69.5	47	6		1980	1908	19
		pTT27	256,992	69.4				251	251	
		pTT8	9322	69				14	14	
*T. thermophilus JL-18*	1.9			69.1	47	6	2	2048	1972	21
		pTTJL1801	265,886	68.5				282	269	13
		pTTJL1802	142,731	68.3				160	155	4
*T. thermophilus SG0.5JP17-16*	1.86			68.9	48	6	2	2007	1924	27
		pTHTHE1601	440,026	67.4				445	429	16
*T. thermophilus ATCC 33923*	2.15			69.4	46	3	1	2337	2253	34

Number of rRNAs represents the number of operons (5S-16S-23S). NCBI, National Center for Biotechnology Information; tRNA, transfer RNA; rRNA, ribosomal RNA.

### Taxonomic position of Thermus sp. NMX2.A1

**Figure 1:**
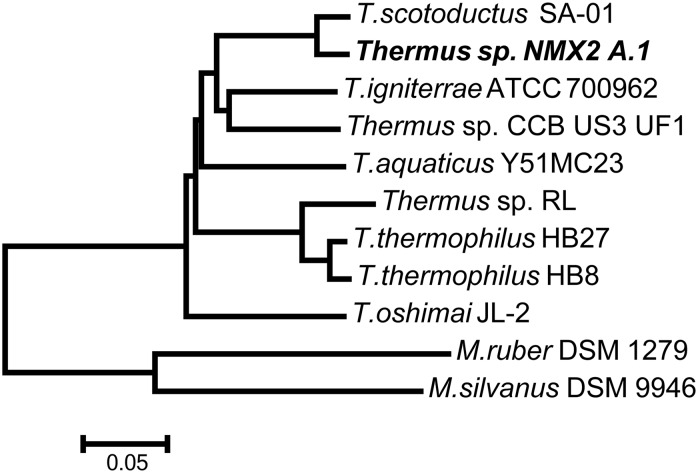
Neighbor-Joining tree designed based on the superalignment of amino acid sequences of 1389 orthologous genes in given bacterial genomes. The phylogenetic position of the strain *Thermus* sp. NMX2 A.1 is highlighted.

The resulting 1389 clusters of orthologous proteins from the BLASTP search over available *Thermus* and *Meiothermus* genomes were used in MUSCLE alignments. The MUSCLE alignments were concatenated into a superalignment of 377,241 amino acid residues and this alignment was used to construct a Neighbor-Joining phylogenetic tree in MEGA 6.0. The resulting phylogenetic tree (Figure 1) illustrates that *Thermus* sp. NMX2 A.1 clusters together with *T. scotoductus* SA-01, which was the closest species in terms of phylogeny. The close evolutionary relationship and metabolic parallels between the two strains has previously been recognized, despite the fact that neither strain’s genome data were available at that point in time (Kieft *et al.* 1999; Balkwill *et al.* 2004). 

### Comparative genome features

**Figure 2:**
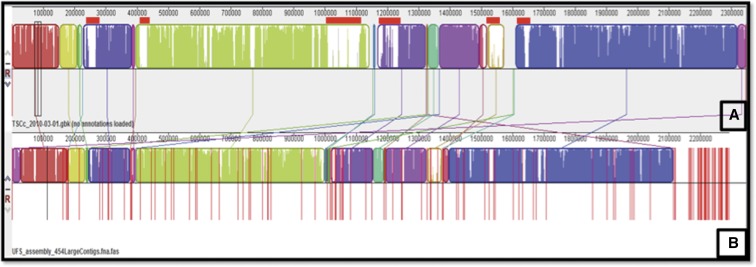
Alignment of the contigs of *Thermus* NMX2 (B) against the *T. scotoductus* SA-01 (A) chromosome by Mauve 2.3.1. Red bars indicate genomic islands predicted in the SA-01 genome by SWGIS (SeqWord Gene Island Sniffer).

The current genome of *Thermus* sp. NMX2 A.1 comprises 132 contigs (Genbank accession number: ATNI00000000.1). MAUVE was used to order the contigs of the *Thermus* sp. NMX2 A.1 using the chromosome of *T. scotoductus* SA-01 as the template (Figure 2).

There are many similarities between *T. scotoductus* SA-01 (A) and *Thermus* sp. NMX2 A.1 (B). The boundaries of the different colored blocks indicate break points in the chromosomes while the vertical red lines indicate contig boundaries. A high degree of synteny is observed. Additionally, the red blocks in the depiction of the *T. scotoductus* SA-01 chromosome indicate genomic islands acquired by horizontal gene transfer (Gounder *et al.* 2011), which were absent in the contigs of the genome of *Thermus* sp. NMX2 A.1. Finally, *Thermus* sp. NMX2 A.1 also has genes (toward the extreme right of the *Thermus* sp. NMX2 A.1 depiction) which are not present within the *T. scotoductus* SA-01 chromosome. These may be either genomic islands acquired by *Thermus* sp. NMX2 A.1 or the genes that were lost by SA-01 after separation of the two lines from the common ancestor (Gounder *et al.* 2011).

The Bidirectional BLAST (Bi-Di BLAST) results (Supplemental Material, Table S1) indicate the comparison of the forward and reverse ORFs of *Thermus* sp. NMX2 A.1 against the selected related strains. An inspection of Table S1 shows that *Thermus* sp. NMX2 A.1 is highly similar to *T. scotoductus* SA-01, as already illustrated by MAUVE and phylogenetic analysis (see Figure 1 and Figure 2). 

A total of 1153 genes are shared by all the *Thermus* strains used in the analysis. *Thermus* sp. NMX2 A.1 contains unique genes not present in any of the other *Thermus* strains used in the analysis. All the genes (54 in total) that were unique to *Thermus* sp. NMX2 A.1 were annotated as hypothetical proteins, except for a Nitrate ABC transporter (TNMX_11560). Of special interest are 12 ORFs (TNMX_12205 to TNMX_RS11770) that form a gene cluster encoding for putative enzyme homologs of the CBB pathway (Figure S2). Although these CBB genes were identified in other *Thermus* strains, it was surprisingly not observed in the closely related *T. scotoductus* SA-01.

A search for horizontally transferred genomic islands using SWGIS predicted only one genomic island of seven hypothetical genes in the contig 123. DNA similarity search through Pre_GI revealed similar genes in the genomic island #2 in the genome of *Cellvibrio gilvus* ATCC 13127 (NC_015671). In the latter genome, all these genes were annotated as heavy metal translocation proteins. Finding only one genomic island was unexpected as 8.3% of the ORFs in *Thermus* sp. NMX2 A.1 had no homologs in the NCBI nonredundant database. However, it is known that *Thermus* organisms are prone to exchange of genetic material by uptake of naked DNA molecules from the environment, especially the genes of closely related bacteria of *Themus*/*Meiothermus* (Kumwenda *et al.* 2014). It is possible that the prediction methods did not detect putative alien genes because the genome was only partially assembled or because of amelioration if the genes were acquired a long time ago.

### Genes involved in DNA uptake

Some *Thermus* strains have been shown to possess natural competence and are able to take up both linear and circular DNA with high efficiency (Friedrich *et al.* 2003; Murugapiran *et al.* 2013). This was also demonstrated for *T. scotoductus* SA-01 (Gounder *et al.* 2011). *Thermus* sp. NMX2 A.1, just like *T*. *scotoductus* SA-01, contains all the homologs of DNA transport genes, suggesting that *Thermus* sp. NMX2 A.1 is naturally competent. These include the genes that encode homologs for: PilC (TNMX_02375), PilD (TNMX_02985), PilF (TNMX_03090), PilM (TNMX_08400), PilN (TNMX_08405), PilO (TNMX_08410), PilQ (TNMX_08420), and PilW (TNMX_08415). Similar to *T. scotoductus* SA-01, the NMX2.A1-strain does not contain the gene encoding for PilA, which is present in *T. thermophilus* HB27 (Gounder *et al.* 2011). In addition, genes encoding for homologs of competence proteins ComEA (TNMX_10095), ComEC (TNMX_10100), ComF (TNMX_06510), DprA (TNMX_09235), and FimA (TNMX_04555) were also identified in *Thermus* sp. NMX2.A1. Bi-Di BLAST analyses of 17 sequenced *Thermus* genomes also revealed that the majority of genes involved with DNA uptake are present in all 17 genomes. Twelve of these DNA uptake genes share at least a 75% protein identity across all the above-mentioned genomes. When surveying the core proteome (75% protein identity cut off for all proteins) of the 17 strains, the proteins responsible for natural competence are present. This indicates that natural competence in the *Thermus* genus is strongly conserved and will undoubtedly afford these bacteria the opportunity to potentially expand their metabolic repertoire.

### Genes involved in denitrification

Several studies have illustrated the partial denitrification pathways in different strains of *Thermus* (Cava *et al.* 2008; Gounder *et al.* 2011; Murugapiran *et al.* 2013; Paraiso and Hedlund 2013; Alvarez *et al.* 2014). In most *Thermus* strains, the genes required in the first step of the denitrification process (reducing nitrate to nitrite) are encoded on a self-transferable DNA element called the Nitrate respiration Conjugation Element (NCE). The NCE encodes the complete nitrate respiratory apparatus comprising two hetero-tetrameric enzymes, NrC and Nar, as well as the regulatory components required for their expression under anoxic conditions in the presence of nitrate (Cava and Berenguer 2006). 

Like a large number of *Thermus* strains, *Thermus* sp. NMX2.A1 also displays a truncated denitrification phenotype, terminating in the production of nitrous oxide instead of dinitrogen. This is probably due to the absence of a periplasmic nitrous oxide reductase (NosZ) (Gounder *et al.* 2011; Murugapiran *et al.* 2013; Alvarez *et al.* 2014). Bi-Di BLAST analyses revealed that *Thermus* sp. NMX2.A1, like the very closely related *T. scotoductus* SA-1, possesses a complete *nar* operon (*nar*GHJIKT) with high sequence identity between the two operons (> 90%). Seven other *Thermus* strains [*T. igniterrae* ATCC700962, both *T. oshimai* strains (DSM12092 and JL-2), *T. antranikianii*, *T. thermophilus* YIM77409, *Thermus* sp 2.9, and two strains of *T. scotoductus* (DSM8553 and DSM21543)] all have complete *nar* operons with similarly high sequence identities (all > 75%) when compared to *Thermus* sp. NMX2.A1. All 17 strains’ genomes encode for the membrane-bound nitrate reductase NarG catalytic subunit homolog that is responsible for nitrate reduction, but with varying sequence identities in comparison to the *Thermus* sp. NMX2.A1 NarG (TMNX_11710) (Cava *et al.* 2008; Alvarez *et al.* 2014). All 17 strains also contain the NarC homolog, a cytochrome *c*-containing nitrate reductase that allows the transfer of electrons from menaquinol to extracellular acceptors when intracellular nitrate becomes scarce (Cava *et al.* 2008; Alvarez *et al.* 2014).

Genes encoding homologs of nitrite respiration proteins NirD (large subunit) and NirJ appear to be present in all 17 sequenced genomes, including NMX2.A1, albeit differently dispersed among the strains. Strikingly, the small subunit of NirD is only found in *Thermus* sp. NMX2.A1 (TNMX_11570) and *T. thermophilus* SG0.5JP17-16, albeit with a low sequence identity of 32%. DELTA-BLAST using TNMX_11570 as the query indicated that the closest match (68% identity) was with a protein containing a Rossman fold NAD(P)^+^ binding site from *Deinococcus deserti*. Interestingly, the simultaneous presence of homologs of cytochrome *cd*_1_ nitrite reductase (NirS) and copper nitrite reductase (NirK) could only be found in *T. oshimai* JL-2 and DSM12092, *T. scotoductus* SA-01, and *Thermus* sp. NMX2.A1. The reduction of nitrite in bacteria is performed by either NirK or NirS, but not both (Murugapiran *et al.* 2013). Thus, the presence of both NirS and NirK homologs in the above-mentioned denitrificant *Thermus* strains is exceptional (Gounder *et al.* 2011; Alvarez *et al.* 2014).

As previously mentioned, *Thermus* sp. NMX2.A1 displayed an incomplete denitrification phenotype that terminated with the formation of nitrous oxide. The formation of nitrous oxide from nitric oxide is facilitated by a heterotrimeric nitric oxide reductase (large subunit NorB and two small subunits NorC and NorH). NorB, NorC, and NorH are present in *Thermus* sp. NMX2A.1 (TNMX_05775, TMNX_05780, and TNMX_05770) as well as *T. thermophilus* (strains JL-18, SG0.5JP17-16, and YIM77409), *Thermus* sp. 2.9, *T. scotoductus* SA-01, and both strains of *T. oshimai* (JL-2 and DSM12092). 

The current genome data of sequenced *Thermus* strains highlight the variability of denitrification and that incomplete denitrification pathways appear to be common phenotypes in *Thermus*. Given the fact that *Thermus* is naturally competent, the variability in denitrification phenotype is indicative of a dispensable *Thermus* genome.

### Genes involved with pigment metabolism

A common characteristic of several *Thermus* strains is their yellow pigmentation due to β-carotene biosynthesis. β-carotene and its hydroxylated products have been implicated in membrane stability and DNA protection from UV-radiation in *Thermus* (Yokoyama *et al.* 1995; Tian and Hua 2010). It is now known that the initial oxidized products of β-carotene in *Thermus* are produced with the aid of a cytochrome P450 monooxygense (CYP450) (Momoi *et al.* 2006). Bi-Di BLAST analyses showed that 15 out of the 17 strains had a CYP450 homolog; only *T. scotoductus* strains SA-01 and DSM8553 (also known as strain SE-1) did not possess a CYP450 homolog. This result seems plausible given where these strains were isolated: the SA-01 strain was isolated in a mine 3.2 km below the surface (Kieft *et al.* 1999) and strain DSM8553 was isolated from tap water (Kristjánsson *et al.* 1994). 

The native electron transport system for CYP175A1 in *T*. *thermophilus* HB27, namely a ferredoxin NAD(P)+ reductase and [3Fe-4S], [4Fe-4S] ferredoxin has been described and shown to effectively shuttle electrons to CYP175A1 to catalyze the hydroxylation of β-carotene (Mandai *et al.* 2009). 

*Thermus* sp. NMX2A.1 possesses homologs of [3Fe-4S], [4Fe-4S] ferredoxin (TNMX_07470), and ferredoxin NAD(P)+ reductase (locus tag: TNMX_01055) proteins that display 99% and 91% identity, respectively, toward the known *T*. *thermophilus* HB27 electron transport proteins.

Based on DELTA-BLAST results, it would seem as if *Thermus* species typically harbor one cytochrome P450 monooxygenase gene. The genome of *Thermus* sp. NMX2A.1 revealed two cytochrome P450 monooxygenase gene homologs (TNMX_06310 and TNMX_12660, partially sequenced) that displayed 98% and 63% amino acid identity, respectively, when compared to CYP175A1. Based on the high amino acid sequence identity and the fact that the substrate binding sites (Yano *et al.* 2003) for both CYP175A1 and TNMX_06310 are identical, it is plausible to suggest that TNM_06310 also encodes a putative β-carotene hydroxylase.

### Genes involved in carbon fixation

The CBB cycle is a series of enzyme-catalyzed reactions responsible for the fixing of carbon. The limiting reaction in this cycle is carried out by the multimeric enzyme ribulose 1,5 bisphosphate carboxylase/oxygenase or ‘rubisco’, which catalyzes the first major step of carbon fixation in the CBB cycle: a process in which ribulose 1,5 bisphosphate reacts with atmospheric CO_2_ and H_2_O to form two molecules of 3-phosphoglycerate (Badger and Price 2003; McNevin *et al.* 2007). The ability to perform this reaction is the defining step in identifying the presence of the CBB-cycle in most organisms. Multimeric rubisco (comprising large and small subunits) is found in plants and most autotrophic organisms, photosynthetic bacteria, algae, and even in some archaea (Andersson 2008; Ichikawa *et al.* 2008).

Homologs of CBB cycle genes, including type 1 rubisco, were identified in a gene cluster of *Thermus* sp. NMX2A.1. A search through the Pre_GI database for sequence similarity with known genomic islands revealed a similar syntenic operon in the genomic island #6 on the second chromosome of the genome of *Ralstonia eutropha* H16 (NC_008314). However, in contrast to *Ralstonia*, DNA composition comparison showed that these genes were native for *Thermus* genomes and most likely this operon in NMX2A.1 emerged in result of recombination of genes with other *Thermus* organisms by DNA uptake (Kumwenda *et al.* 2014).

Bi-Di BLAST analyses indicated that, excluding rubisco, the putative CBB cycle enzyme homologs could be identified in all 17 *Thermus* strains, except for one enzyme that is responsible for regenerating ribulose-1,5-bisphosphate from ribulose-5-phosphate, namely phosphoribulokinase (PRK). Pathway Tools was also unable to annotate a PRK to complete the CBB-cycle (Figure S1). A survey of several *Thermus* genomes revealed two adjacent ribulose-phosphate 3-epimerases in the CBB-gene cluster (Figure S2) and further Pathway Tools analyses indicated that one of these is the enzyme responsible for the reaction 2.7.1.19 usually catalyzed by the PRK (Figure S1). DELTA-BLAST using the smaller of the two epimerases from *Thermus* sp. NMX2.A1 (TNMX_12225) produced at least 500 positive results for ribulose-phosphate 3-epimerase from a wide variety of bacteria. When the larger epimerase (TNMX_12230) was subjected to the same DELTA BLAST, it showed that about 3% of the results were for ribulose-phosphate 3-epimerases containing a conserved protein domain called nucleoside/nucleotide kinase (NK), which were almost exclusively confined to *Meiothermus*/*Thermus* strains. Strikingly, the remaining BLAST results were for PRKs from various bacteria and cyanobacteria. TNMX_12230 was also analyzed in the BRENDA enzyme information system and was surprisingly identified as a PRK. BRENDA also identified THEOS_RS08585 and N686_RS06455, from *T*. *oshimai* JL-2 and *T*. *scotoductus* K12, respectively (Figure S2), as PRKs. The results are indicative of a possible misannotation of these proteins as epimerases instead of putative PRKs. However, given the location of TNMX_12230 and other NK-region-containing epimerases in the CBB-gene cluster and the BLAST results, it could be argued that these epimerases are possibly PRKs, a crucial enzyme for regenerating the sugar substrate for rubisco and thus completing the CBB-cycle.

**Figure 3:**
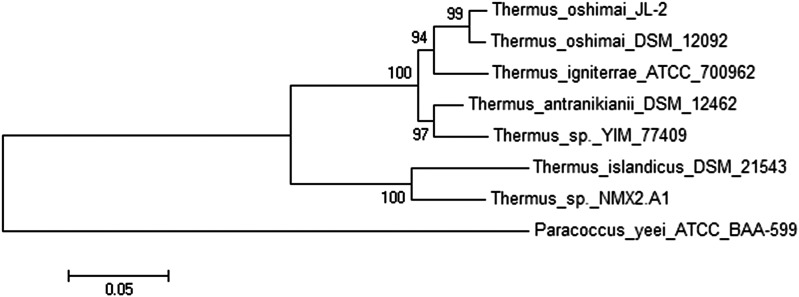
Consensus phylogenetic tree of selected ribulose bisphosphate carboxylase small chain (EC 4.1.1.39), ribulose bisphosphate carboxylase large chain (EC 4.1.1.39), and the fructose-1,6-bisphosphate, GlpX type (EC 3.1.3.11) from *Thermus* spp. rooted with an outlier.

Bi-Di BLAST (Table S1) indicated that the rubisco homolog is not ubiquitously found in all species of *Thermus*. For example, the rubisco homolog is not present in any *T. thermophilus* strain except for *T. thermophilus* YIM77409. As for the *T. scotoductus* strains, Bi-Di BLAST analyses revealed that both rubisco subunits are present in *T*. *scotoductus* DSM21543 but that *T. scotoductus* K12 only possesses the large rubisco subunit (Figure S2). No homologs of the rubisco subunits were identified in *T*. *scotoductus* strain DSM8553 and SA-01. The absence of rubisco in the latter strain was surprising, given the very close similarity displayed between *Thermus* sp. NMX2.A1 and *T. scotoductus* SA-01 on protein level (Figure 1). Figure 3 shows the phylogenetic analysis of the artificially concatenated amino acid sequence containing the two ribulose bisphosphate carboxylase subunits and the fructose-1,6-bisphosphate, GlpX type proteins. 

These CBB-cycle proteins were selected for phylogenetic analyses due to their adjacent gene topology and orientation on the selected *Thermus* genomes (Figure S2). An evolutionary relationship between the proteins from *Thermus* sp. NMX2.A1 and the six other *Thermus* proteins is evident. Interestingly, a close evolutionary relationship with the proteins from *T. islandicus* DSM 21543 is observed with that from *Thermus* sp. NMX2.A1.

The *Thermus* strains depicted in Figure 3 were isolated from vastly different geographical locations that range from Iceland, Portugal, and the USA (Hudson *et al.* 1989; Williams *et al.* 1996; Chung *et al.* 2000; Bjornsdottir *et al.* 2009). Reviewing the environments from which these *Thermus* strains were isolated could illuminate the reasons for the presence of these carboxylases in a few *Thermus* strains. Table S2 and Table S3 contain some geographical and physico-chemical data of sites from which rubisco-containing *Thermus s*trains were isolated. Although the hot spring water these various *Thermus* spp. reside in are from widespread geographical locations, there seems to be a common thread: appreciable amounts of HCO_3_^-^ and CO_2_ coupled with water pH ranging from neutral to alkaline. The HCO_3_^-^ in these hot springs has its origin from various carbonates in rocks, sediments, and soils that are heated from ultradeep geothermal activity, biogenic CO_2_ that dissolves in water, or even CO_2_ escaping from the soil (McGee *et al.* 2006). It would seem as if *Thermus* strains that harbor rubisco genes are isolated from hot springs that have readily available HCO_3_^-^. Further supporting this hypothesis is that *T. thermophilus* HB27, *T. thermophilus* HB8, and *T. scotoductus* SA-01 were all isolated from environments with very low levels of HCO_3_^-^ and do not contain the complete CBB cycle, having never had the need to acquire them, as was the case for *Thermus* sp. NMX2.A1. Further, *T. scotoductus* SA-01 was isolated from the deep subsurface, an environment where it was shown that the CBB cycle genes were almost completely absent and carbon metabolism occurs predominantly by the reductive acetyl-CoA pathway (Simkus *et al.* 2016).

*Thermus* strains containing the type I rubisco homolog were isolated from hot springs with temperatures that range from 50–88°. These high temperatures decrease oxygenation levels in the water. Given rubisco’s binding preference for O_2_ as opposed to CO_2_, the less oxygenated water could mean that rubisco would be able to bind CO_2_ more freely. This might explain why no homologs for carboxysome proteins were identified for any of the rubisco-containing *Thermus* strains (Table S2). Interestingly, homologs for carbonic anhydrases were identified for most rubisco-containing *Thermus* strains but for none of the rubisco-devoid *Thermus* strains (File S1, Table S2 and Table S3). We speculate that the putative CBB-cycle could be active to fix carbon in rubisco-containing *Thermus* strains in the presence of bicarbonate, carbonic anhydrase, and low oxygenation. Since carbon fixation requires energy, a possible source of energy for chemolitotrophic growth could be the oxidation of sulfur compounds (Gounder *et al.* 2011; Murugapiran *et al.* 2013; Table S1). 

We conclude that rubisco-containing *Thermus* strains can probably fix carbon as chemolitotrophes, and that rubisco-devoid *Thermus* strains are likely obligate chemo-heterotrophs. 

## Supplementary Material

Supplemental Material
