# Supplementary material for: Whole Genome Comparison of Thermus sp. NMX2.A1 Reveals Principal Carbon Metabolism Differences with Closest Relation Thermus scotoductus SA-01
Source: G3 (Bethesda). 2016 Jul 11;6(9):2791–7. doi: 10.1534/g3.116.032953 (PMC5015936; doi:10.1534/g3.116.032953)
Supplement: Supplemental Material [file supp_6_9_2791__index.html]

Whole Genome Comparison of Thermus sp. NMX2.A1 Reveals Principle Carbon Metabolism Differences with Closest Relation Thermus scotoductus SA-01 — Supplemental Material 

# Whole Genome Comparison of *Thermus* sp. NMX2.A1 Reveals Principle Carbon Metabolism Differences with Closest Relation *Thermus scotoductus* SA-01

## Supplemental Material for Müller *et al.*, 2016

**Files in this Data Supplement:**

- Figure S1 - Comparison of the putative Calvin-Benson-Bassham cycles of *Thermus* sp. NMX2.A1 with *Thermus* spp. available in the BioCyc PGDB database using Species Compare in Pathway Tools. (.pdf, 491 KB)
- Figure S2 - Comparison of putative Calvin Benson-Bassham gene topologies of *Thermus* sp. NMX2.A1 with select sequenced *Thermus* strains. (.pdf, 398 KB)
- Table S2 - Geographical and physico-chemical data from isolation sites of rubisco-containing Thermus strains. (.pdf, 182 KB)
- Table S3 - Geographical and physico-chemical data from isolation sites of *Thermus* strains devoid of rubisco. (.pdf, 155 KB)
- File S1 - Supplementary references. (.pdf, 160 KB)
- Table S1 - Genome comparison. (.xlsx, 2 MB)
