# Supplementary material for: Whole Genome Comparison of Thermus sp. NMX2.A1 Reveals Principal Carbon Metabolism Differences with Closest Relation Thermus scotoductus SA-01
Source: G3 (Bethesda). 2016 Jul 11;6(9):2791–7. doi: 10.1534/g3.116.032953 (PMC5015936; doi:10.1534/g3.116.032953)
Supplement: Supplemental Material [file supp_g3.116.032953_FigureS1.pdf]

Organism	Evidence Glyph	Enzymes and Genes for Calvin-Benson-Bassham cycle	
T. sp. NMX2 A.1	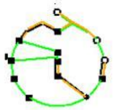	EC 4.1.1.39	ribulose 1,5-bisphosphate carboxylase small subunit: TNMX_12240
			ribulose 1,5-bisphosphate carboxylase: rbcL
		EC 2.7.2.3	phosphoglycerate kinase: pgk
		EC 1.2.1.13	None
		EC 5.3.1.1	triosephosphate isomerase: TNMX_06865
		EC 4.1.2.-	None
		EC 3.1.3.37	None
		EC 2.2.1.1	transketolase: TNMX_12295
		EC 5.3.1.6	ribose 5-phosphate isomerase: TNMX_05945
		EC 4.1.2.13	fructose 1,6-bisphosphate aldolase: TNMX_12220
			fructose 1,6-bisphosphate aldolase: TNMX_12285
		EC 3.1.3.11	fructose 1,6-bisphosphatase: TNMX_041285
			fructose 1,6-bisphosphatase: glpX
			fructose 1,6-bisphosphatase: glpX
		EC 2.2.1.1	transketolase: TNMX_12295
		EC 5.1.3.1	ribulose-phosphate 3-epimerase: GTNMX_12230
			ribulose-phosphate 3-epimerase: TNMX_12225
		EC 2.7.1.19	None

Organism	Evidence Glyph	Enzymes and Genes for Calvin-Benson-Bassham cycle	
T. igniterrae ATCC 700962	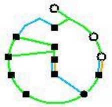	EC 4.1.1.39	ribulose bisphosphate carboxylase small chain: B128_RS0108640
			ribulose bisophosphate carboxylase: rbcL
		EC 2.7.2.3	phosphoglycerate kinase: pgk
		EC 1.2.1.13	glyceraldehyde-3-phosphate dehydrogenase: B128_RS0102675
		EC 5.3.1.1	triosephosphate isomerase: B128_RS0102665
		EC 4.1.2.-	fructose-bisphosphate aldolase: kbaY
		EC 3.1.3.37	fructose 1,6-bisphosphatase: B128_RS0106090
		EC 2.2.1.1	transketolase: B128_RS0107750
		EC 5.3.1.6	ribose 5-phosphate isomerase: B128_RS0104955
		EC 4.1.2.13	fructose-bisphosphate aldolase: kbaY
			fructose-bisphosphate aldolase: kbaY
		EC 3.1.3.11	fructose 1,6-bisphosphatase: B128_RS0108650
			fructose 1,6-bisphosphatase: B128_RS0106090
		EC 2.2.1.1	transketolase: B128_RS0107750
		EC 5.1.3.1	ribulose-phosphate 3-epimerase: B128_RS0108630
			ribulose-phosphate 3-epimerase: B128_RS0108625
		EC 2.7.1.19	ribulose-phosphate 3-epimerase: B128_RS0108630

Organism	Evidence Glyph	Enzymes and Genes for Calvin-Benson-Bassham cycle	
T. islandicus DSM 21543	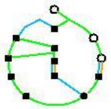	EC 4.1.1.39	ribulose bisphosphate carboxylase small chain: H531_RS0106280
			ribulose bisophosphate carboxylase: rbcL
		EC 2.7.2.3	phosphoglycerate kinase: pgk
		EC 1.2.1.13	glyceraldehyde-3-phosphate dehydrogenase: H531_RS0104990
		EC 5.3.1.1	triosephosphate isomerase: H531_RS0105000
		EC 4.1.2.-	fructose-bisphosphate aldolase: kbaY
		EC 3.1.3.37	fructose 1,6-bisphosphatase: H531_RS0111350
		EC 2.2.1.1	transketolase: H531_RS0106335
		EC 5.3.1.6	ribose 5-phosphate isomerase: H531_RS0105725
		EC 4.1.2.13	fructose-bisphosphate aldolase: kbaY
			fructose-bisphosphate aldolase: kbaY
		EC 3.1.3.11	fructose 1,6-bisphosphatase: H531_RS0106290
			fructose 1,6-bisphosphatase: H531_RS0103400
			fructose 1,6-bisphosphatase: H531_RS0111350
		EC 2.2.1.1	transketolase: H531_RS0106335
		EC 5.1.3.1	ribulose-phosphate 3-epimerase: H531_RS0106270
			ribulose-phosphate 3-epimerase: H531_RS0106265
		EC 2.7.1.19	ribulose-phosphate 3-epimerase: H531_RS0106270

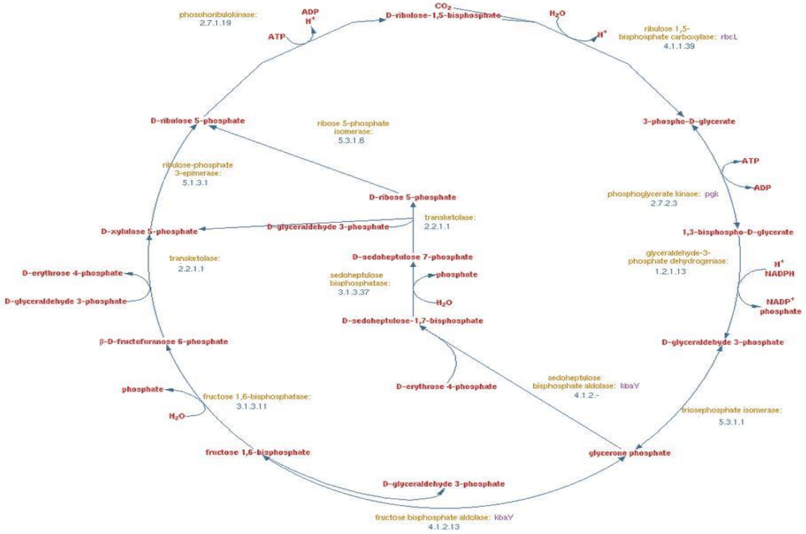

Key to Pathway Evidence Glyph Edge Colors

Green	**Enzyme present.** An enzyme for this reaction **has** been identified in this organism.
Blue	**Enzyme present by hole filler.** An enzyme for this reaction **has** been identified in this organism by the Pathway Hole Filler.
Black	**Enzyme absent.** An enzyme for this reaction **has not** been identified in this organism.
Red	**Key reaction.** The reaction has been designated a key reaction of this pathway
Orange	**Unique reaction.** This reaction has not been designated a key reaction, but is not present in any other pathway in this PGDB (bu
Gray	**Spontaneous/other.** Used for spontaneous reactions, or used for lines that do not denote reactions (e.g., denoting repetition in

Organism	Evidence Glyph	Enzymes and Genes for Calvin-Benson-Bassham cycle	
T. oshimai JL-2	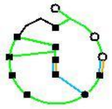	EC 4.1.1.39	ribulose bisphosphate carboxylase small subunit: Theos_1734<br>ribulose 1,5-bisphosphate carboxylase, large subunit: Theos_1735
		EC 2.7.2.3	3-phosphoglycerate kinase: Theos_0920
		EC 1.2.1.13	glyceraldehyde-3-phosphate dehydrogenase, type I: Theos_0919
		EC 5.3.1.1	triosephosphate isomerase: Theos_0953
		EC 4.1.2.-	fructose-1,6-bisphosphate aldolase, class II: Theos_1730
		EC 3.1.3.37	fructose-1,6-bisphosphatase, class II: Theos_1644
		EC 2.2.1.1	transketolase: Theos_1745
		EC 5.3.1.6	ribose 5-phosphate isomerase: Theos_0857
		EC 4.1.2.13	fructose-1,6-bisphosphate aldolase, class II: Theos_0264<br>fructose-1,6-bisphosphate aldolase, class II: Theos_1730
		EC 3.1.3.11	fructose-1,6-bisphosphatase family protein: Theos_0367<br>archaeal fructose 1,6-bisphosphatase: Theos_1016<br>fructose-1,6-bisphosphatase, class II: Theos_1644<br>fructose-1,6-bisphosphatase, class II: Theos_1736
		EC 2.2.1.1	transketolase: Theos_1745
		EC 5.1.3.1	ribulose-phosphate 3-epimerase: Theos_1731
		EC 2.7.1.19	None

Organism	Evidence Glyph	Enzymes and Genes for Calvin-Benson-Bassham cycle	
T. scotoductus SA-01	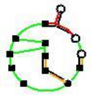		**This pathway is not marked as present in this organism.**
		EC 4.1.1.39	None
		EC 2.7.2.3	phosphoglycerate kinase: pgk
		EC 1.2.1.13	None
		EC 5.3.1.1	triose-phosphate isomerase: tpiA
		EC 4.1.2.-	None
		EC 3.1.3.37	None
		EC 2.2.1.1	transketolase: tkt
		EC 5.3.1.6	ribose 5-phosphate isomerase A: rpiA
		EC 4.1.2.13	fructose-1,6-bisphosphate aldolase: fba
		EC 3.1.3.11	fructose-1,6-bisphosphatase: glpX
		EC 2.2.1.1	transketolase: tkt
		EC 5.1.3.1	ribulose-phosphate 3-epimerase: rpe
		EC 2.7.1.19	phosphoribulokinase/uridine kinase: TSC_c19980

Organism	Evidence Glyph	Enzymes and Genes for Calvin-Benson-Bassham cycle	
T. aquaticus Y51MC23	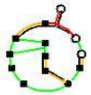		**This pathway is not marked as present in this organism.**
		EC 4.1.1.39	None
		EC 2.7.2.3	phosphoglycerate kinase: pgk
		EC 1.2.1.13	None
		EC 5.3.1.1	triosephosphate isomerase: TAQDRAFT_RS04300
		EC 4.1.2.-	None
		EC 3.1.3.37	None
		EC 2.2.1.1	transketolase: TAQDRAFT_RS03285
		EC 5.3.1.6	ribose 5-phosphate isomerase A: TAQDRAFT_RS02530
		EC 4.1.2.13	fructose-bisphosphate aldolase: kbaY
		EC 3.1.3.11	fructose 1,6-bisphosphatase: TAQDRAFT_RS05270<br>fructose 1,6-bisphosphatase: TAQDRAFT_RS10510
		EC 2.2.1.1	transketolase: TAQDRAFT_RS03285
		EC 5.1.3.1	ribulose-phosphate 3-epimerase: TAQDRAFT_RS03275
		EC 2.7.1.19	None

Organism	Evidence Glyph	Enzymes and Genes for Calvin-Benson-Bassham cycle	
T. caliditerrae	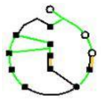	EC 4.1.1.39	ribulose 1,5-bisphosphate carboxylase small subunit: EI72_RS01305<br>ribulose 1,5-bisphosphate carboxylase: rbcL
		EC 2.7.2.3	phosphoglycerate kinase: pgk
		EC 1.2.1.13	None
		EC 5.3.1.1	triosephosphate isomerase: EI72_RS04495
		EC 4.1.2.-	None
		EC 3.1.3.37	None
		EC 2.2.1.1	transketolase: EI72_RS01250
		EC 5.3.1.6	ribose 5-phosphate isomerase: EI72_RS02360
		EC 4.1.2.13	None
		EC 3.1.3.11	fructose 1,6-bisphosphatase: EI72_RS01295<br>fructose 1,6-bisphosphatase: EI72_RS01970
		EC 2.2.1.1	transketolase: EI72_RS01250
		EC 5.1.3.1	ribulose-phosphate 3-epimerase: EI72_RS01315<br>ribulose-phosphate 3-epimerase: EI72_RS01320
		EC 2.7.1.19	None

Organism	Evidence Glyph	Enzymes and Genes for Calvin-Benson-Bassham cycle	
T. filiformis	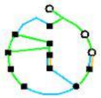	EC 4.1.1.39	ribulose 1,5-bisphosphate carboxylase small subunit: THFILI_RS03165
			ribulose 1,5-bisphosphate carboxylase: rbcL
		EC 2.7.2.3	phosphoglycerate kinase: pgk
		EC 1.2.1.13	glyceraldehyde-3-phosphate dehydrogenase: THFILI_RS07430
		EC 5.3.1.1	triosephosphate isomerase: THFILI_RS07420
		EC 4.1.2.-	tagatose-bisphosphate aldolase: kbaY
		EC 3.1.3.37	fructose 1,6-bisphosphatase: THFILI_RS10045
		EC 2.2.1.1	transketolase: THFILI_RS06125
		EC 5.3.1.6	ribose 5-phosphate isomerase: THFILI_RS04005
		EC 4.1.2.13	tagatose-bisphosphate aldolase: kbaY
		EC 3.1.3.11	fructose 1,6-bisphosphatase: THFILI_RS10045
			fructose 1,6-bisphosphatase: THFILI_RS08190
			fructose 1,6-bisphosphatase: THFILI_RS03155
		EC 2.2.1.1	transketolase: THFILI_RS06125
		EC 5.1.3.1	ribulose-phosphate 3-epimerase: THFILI_RS03175
			ribulose-phosphate 3-epimerase: THFILI_RS03180
		EC 2.7.1.19	ribulose-phosphate 3-epimerase: THFILI_RS03175

Organism	Evidence Glyph	Enzymes and Genes for Calvin-Benson-Bassham cycle	
T. sp. CCB_US3_UF1	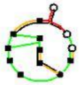		**This pathway is not marked as present in this organism.**
		EC 4.1.1.39	None
		EC 2.7.2.3	phosphoglycerate kinase: TCCBUS3UF1_11130
		EC 1.2.1.13	glyceraldehyde-3-phosphate dehydrogenase: TCCBUS3UF1_11120
		EC 5.3.1.1	triosephosphate isomerase: TCCBUS3UF1_11140
		EC 4.1.2.-	None
		EC 3.1.3.37	None
		EC 2.2.1.1	transketolase: TCCBUS3UF1_17960
		EC 5.3.1.6	Ribose 5-phosphate isomerase: TCCBUS3UF1_15860
		EC 4.1.2.13	Fructose-bisphosphate aldolase: TCCBUS3UF1_3550
			Fructose-bisphosphate aldolase: TCCBUS3UF1_20000
		EC 3.1.3.11	Fructose-1,6-bisphosphatase, class II: TCCBUS3UF1_16590
		EC 2.2.1.1	transketolase: TCCBUS3UF1_17960
		EC 5.1.3.1	Ribulose-5-phosphate 3-epimerase: TCCBUS3UF1_17940
		EC 2.7.1.19	None

Organism	Evidence Glyph	Enzymes and Genes for Calvin-Benson-Bassham cycle	
T. thermophilus HB27	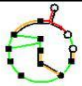		**This pathway is not marked as present in this organism.**
		EC 4.1.1.39	None
		EC 2.7.2.3	2-phosphoglycerate kinase: TTC0121
			phosphoglycerate kinase: pgk
		EC 1.2.1.13	None
		EC 5.3.1.1	triosephosphate isomerase: tpiA
		EC 4.1.2.-	None
		EC 3.1.3.37	None
		EC 2.2.1.1	transketolase: TTC1896
		EC 5.3.1.6	ribose-5-phosphate isomerase A: TTC0932
		EC 4.1.2.13	fructose-bisphosphate aldolase: TTC1414
		EC 3.1.3.11	fructose 1,6-bisphosphatase II: glpX
		EC 2.2.1.1	transketolase: TTC1896
		EC 5.1.3.1	ribulose-phosphate 3-epimerase: TTC1898
		EC 2.7.1.19	None

Organism	Evidence Glyph	Enzymes and Genes for Calvin-Benson-Bassham cycle	
T. thermophilus HB8	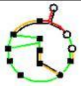		**This pathway is not marked as present in this organism.**
		EC 4.1.1.39	None
		EC 2.7.2.3	phosphoglycerate kinase: pgk
		EC 1.2.1.13	glyceraldehyde 3-phosphate dehydrogenase: TTHA0905
		EC 5.3.1.1	triosephosphate isomerase: tpiA
		EC 4.1.2.-	None
		EC 3.1.3.37	None
		EC 2.2.1.1	transketolase: TTHA0108
		EC 5.3.1.6	ribose-5-phosphate isomerase A: TTHA1299
		EC 4.1.2.13	fructose-1,6-bisphosphate aldolase: TTHA1773
		EC 3.1.3.11	fructose 1,6-bisphosphatase II: glpX
		EC 2.2.1.1	transketolase: TTHA0108
		EC 5.1.3.1	ribulose-phosphate 3-epimerase: TTHA0106
		EC 2.7.1.19	None

Organism	Evidence Glyph	Enzymes and Genes for Calvin-Benson-Bassham cycle	
T. thermophilus JL-18	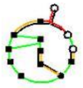		**This pathway is not marked as present in this organism.**
		EC 4.1.1.39	None
		EC 2.7.2.3	3-phosphoglycerate kinase: TtJL18_1141
		EC 1.2.1.13	None
		EC 5.3.1.1	triosephosphate isomerase: TtJL18_1110
		EC 4.1.2.-	None
		EC 3.1.3.37	None
		EC 2.2.1.1	transketolase: TtJL18_1811
		EC 5.3.1.6	ribose 5-phosphate isomerase: TtJL18_0751
		EC 4.1.2.13	fructose-1,6-bisphosphate aldolase: TtJL18_0271
		EC 3.1.3.11	fructose-1,6-bisphosphatase: TtJL18_0601
			fructose 1,6-bisphosphatase: TtJL18_1069
			inositol monophosphatase/fructose-1,6-bisphosphatase family protein: TtJL18_1777
		EC 2.2.1.1	transketolase: TtJL18_1811
		EC 5.1.3.1	ribulose-phosphate 3-epimerase: TtJL18_1809
		EC 2.7.1.19	None

Organism	Evidence Glyph	Enzymes and Genes for Calvin-Benson-Bassham cycle	
T. thermophilus SG0.5JP17-16	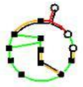		**This pathway is not marked as present in this organism.**
		EC 4.1.1.39	None
		EC 2.7.2.3	phosphoglycerate kinase: Ththe16_0909
		EC 1.2.1.13	None
		EC 5.3.1.1	triosephosphate isomerase: Ththe16_0938
		EC 4.1.2.-	None
		EC 3.1.3.37	None
		EC 2.2.1.1	transketolase: Ththe16_0277
		EC 5.3.1.6	ribose 5-phosphate isomerase: Ththe16_1310
		EC 4.1.2.13	fructose-1,6-bisphosphate aldolase: Ththe16_1790
		EC 3.1.3.11	fructose-1,6-bisphosphatase: Ththe16_1457
		EC 2.2.1.1	transketolase: Ththe16_0277
		EC 5.1.3.1	ribulose-phosphate 3-epimerase: Ththe16_0279
		EC 2.7.1.19	None

Fig. S1 Comparison of the putative Calvin-Benson-Bassham cycles of *Thermus* sp. NMX2.A1 with *Thermus* spp. available in the BioCyc PGDB database using Species Compare in Pathway Tools.
