# Supplementary material for: Whole Genome Comparison of Thermus sp. NMX2.A1 Reveals Principal Carbon Metabolism Differences with Closest Relation Thermus scotoductus SA-01
Source: G3 (Bethesda). 2016 Jul 11;6(9):2791–7. doi: 10.1534/g3.116.032953 (PMC5015936; doi:10.1534/g3.116.032953)
Supplement: Supplemental Material [file supp_g3.116.032953_FigureS2.pdf]

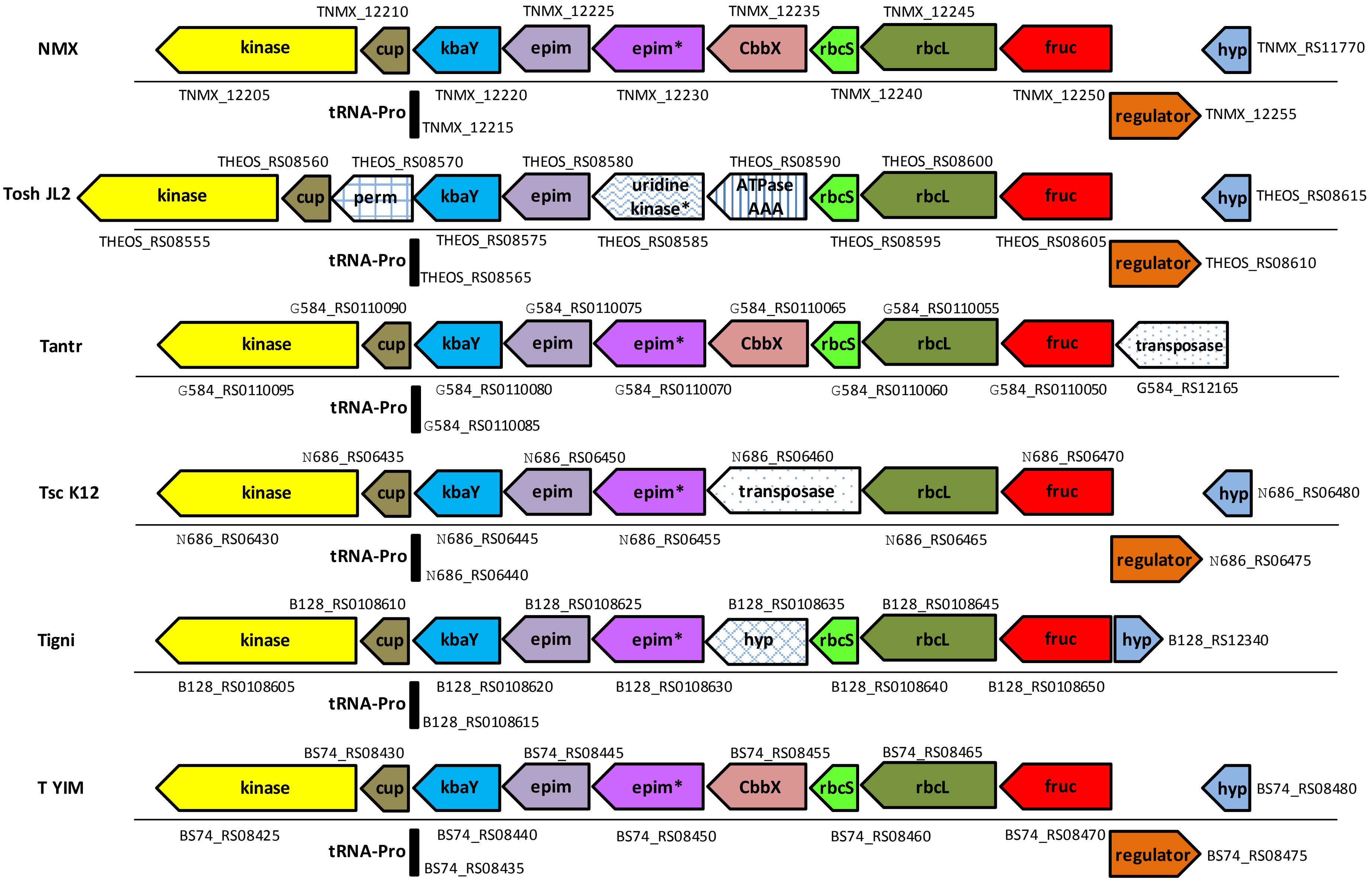

Fig. S2 Comparison of putative Calvin Benson-Bassham gene topologies of *Thermus* sp. NMX2.A1 with select sequenced *Thermus* strains

Figure key:

Genes encoding for:

- kinase = histidine kinase
- cup = cupin region containing protein
- kbaY = tagatose-bisphosphate aldolase
- epim = ribulose-phosphate 3-epimerase
- cbbX = rubsico expression protein CbbX
- rbcS = ribulose 1,5-bisphosphate carboxylase small subunit
- rbcL = ribulose 1,5-bisphosphate carboxylase large subunit
- fruc = fructose 1,6-bisphosphatase
- regulator = LysR family transcriptional regulator
- hyp = hypothetical protein
- perm = glycerol transporter permease
- * containing a conserved nucleoside/nucleotide kinase (NK) protein domain

Thermus strain and genome/contig used:

- NMX = *Thermus* sp. NMX2.A1 – (NZ_ATNI01000119.1)
- Tosh JL2 = *Thermus oshimai* JL-2 – (NC_019386.1)
- Tantr = *Thermus antranikianii – (*NZ_AUIW01000018.1)
- Tsc K12 *= Thermus scotoductus* – K12 – (NZ_JQLJ01000001.1)
- Tigni = *Thermus igniterrae* – (NZ_AQWU01000067.1)
- T YIM= *Thermus* sp. YIM 77409 (synonym: *Thermus amyloliquefaciens)* – (NZ_JQMV01000003.1)
