# Supplementary material for: Whole Genome Comparison of Thermus sp. NMX2.A1 Reveals Principal Carbon Metabolism Differences with Closest Relation Thermus scotoductus SA-01
Source: G3 (Bethesda). 2016 Jul 11;6(9):2791–7. doi: 10.1534/g3.116.032953 (PMC5015936; doi:10.1534/g3.116.032953)
Supplement: Supplemental Material [file supp_g3.116.032953_TableS2.pdf]

TABLE S2 Geographical and physico-chemical data from isolation sites of rubisco-containing *Thermus* strains

Strain	Isolated from	Rubisco large and small subunit present?	Carbonic anhydrase or Ferripyochelin binding protein[a] present?	Carboxysome proteins (carbon dioxide concentrating mechanism protein)	Temperature (°C) and pH of isolation point	Prevalent geochemical trait of hot spring / thermal area	References
Thermus sp. YIM 77409	Sediment from Niujiea ancient hot spring in Eryuan, Dali, Yunnan Province, South-West China	Yes	No DELTA BLAST result for either Carbonic anhydrase or Ferripyochelin binding protein	No[b]	T = 84<br>pH 7.4	Na^+ = 206.3 mg/L<br>NH_4^+ = 115. 94 mg/L<br>K^+ = 38.51 mg/L<br>Ca^{2+} = 37.06 mg/L<br>NO_3^-= 8.47 mg/L<br>NO_2^-= 2.32 mg/L	Personal communication: Prof. Brian Hedlund (University of Nevada, USA)
T. antranikianii	Hot water spring, Hruni, Iceland	Yes	Carbonic anhydrase	No	T = 80 – 88<br>pH 9.1	SiO_2 = 54.9 mg/L<br>Na^+ = 39.8 mg/L<br>SO_4^{2-} = 28.3 mg/L<br>HCO_3^- = 27.35 mg/L<br>Cl^- = 12.9 mg/L<br>Ca^{2+} = 4.01 mg/L<br>K^+ = 0.6 mg/L	(1)<br>Personal communication: Magnús Ólafsson, Iceland Geosurvey
T. scotoductus KI2[c]	Biofilm community on trunks of dead, steaming trees in the Puhimau thermal area on the flank of Kilauea volcano, Hawaii, USA.	No[a]	Carbonic anhydrase	No	T = 55 - 66<br>pH 3.8 – 4.2	Escaping steam and dead vegetation trademark of this thermal area<br>High levels of CO_2, Hg, He and various sulfur compounds rising from ground	(2)<br>Personal communication: Prof. Gary King (Louisiana State University, USA)
Thermus sp. NMX2A.1	Jemez Hotspring, New Mexico, USA	Yes	Ferripyochelin binding protein	No	T = 71<br>pH = 7.7	Saturated in $CaCO_3$<br>Travertine is pure calcite (no aragonite present)	(3)<br>Personal communication: Prof. Tom Kieft (New Mexico Tech, USA)

Strain	Isolated from	Rubisco large and small subunit present?	Carbonic anhydrase or Ferripyochelin binding protein[a] present?	Carboxysome proteins (carbon dioxide concentrating mechanism protein)	Temperature (°C) and pH of isolation point	Prevalent geochemical trait of hot spring / thermal area	References
T. filiformis	Clamshell hotspring, Waimangu, New Zealand	Yes	Carbonic anhydrase	No	T = 85<br>pH = 8.0	Na^{+} = 729 mg/L<br>Cl^{-} = 528 mg/L<br>Si^{4+} = 301 mg/L<br>HCO_3^{-} = 174 mg/L<br>SO_4^{2-} = 135 mg/L<br>K^{+} = 100 mg/L	(1, 4)<br>Personal communication: Prof. Hugh Morgan (University of Waikato, New Zealand)
T. islandicus	Fumarole HS 605 in Torfajokull geothermal area, Iceland	Yes	Ferripyochelin binding protein	No	T = 74<br>pH = 6.0	Emitting large volumes gas:<br>CO_2 = 8330 mmole/kg<br>H_2 = 47.9 mmole/kg<br>H_2S = 14.5 mmole/kg<br>Hot spring waters in this area are of the bicarbonate type	(5 - 7)
T. yunnanensis	Hot spring locations of Tengchong, Yunnan province, (China)	Yes	Carbonic anhydrase	No	Not reported	Not reported	(8)
T. igniterrae	Hot water spring, Reykyaflot Iceland	Yes	Ferripyochelin binding protein	No	T = 73<br>pH = 8.9	SiO_2 = 252.4 mg/L<br>Na^{+} = 109.1 mg/L<br>SO_4^{2-} = 64.1 mg/L<br>HCO_3^{-} = 54.4 mg/L<br>Cl^{-} = 30.7 mg/L<br>K^{+} = 5.97 mg/L<br>Ca^{2+} = 1.52 mg/L	(1)<br>Personal communication: Magnús Ólafsson, Iceland Geosurvey

Strain	Isolated from	Rubisco large and small subunit present?	Carbonic anhydrase or Ferripyochelin binding protein[a] present?	Carboxysome proteins (carbon dioxide concentrating mechanism protein)	Temperature (°C) and pH of isolation point	Prevalent geochemical trait of hot spring / thermal area	References
T. oshimai<br>*T. oshimai* JL-2	Hot springs from S. Pedro do Sul, (Portugal) and Hveragerdi, (Iceland)<br>Sandy's Spring West (Nevada, USA)	Yes	Carbonic anhydrase	No	T = 50.0[e]<br>pH = 8.5[e]<br>T = 86.6[f]<br>pH = 7.21[f]	Iceland: Mainly Na-HCO_3 and Na-Cl type water<br>USA: Circumneutral Na-Cl springs, high levels of SiO_2, Na^+ and Cl^-	(1, 9 - 12)
T.caliditerrae	Hot spring from Hydrothermal Explosion (Shuirebaozhaqu), Rehai geothermal field in Tengchong county (south-western China)	Yes	Carbonic anhydrase	No	T = 79.8<br>pH = 7.5	Spring water of Na-HCO_3-Cl type, Major anion is HCO_3^- (622 mg/L)	(13 - 15)

[a]Gamma-carbonic anhydrase-like protein

[b]No DELTA BLAST results were obtained for carbon dioxide concentrating mechanism protein (ccm) when using either the ccmK, K2, K4, L, M,N and O-proteins from of model cyanobacterium *Synechococcus elongatus* PCC *7942* as query

[c]*T. scotoductus* KI2: partial genome sequence. Also note that this Rubisco amino acid sequence is incomplete on the C-terminal end.

[d]DELTA-BLAST did not produce a result for the small Rubisco-subunit of strain KI2

[e]*T. oshimai* strain JL-2 isolated from hotspring in Nevada, USA

[f]*T. oshimai* type strain isolated from hotspring off run at S. Pedro do Sul, Portugal. Hot spring vent was 69°C, pH 8.8 (personal communication Milton da Costa)
