# Supplementary material for: Whole Genome Comparison of Thermus sp. NMX2.A1 Reveals Principal Carbon Metabolism Differences with Closest Relation Thermus scotoductus SA-01
Source: G3 (Bethesda). 2016 Jul 11;6(9):2791–7. doi: 10.1534/g3.116.032953 (PMC5015936; doi:10.1534/g3.116.032953)
Supplement: Supplemental Material [file supp_g3.116.032953_TableS3.pdf]

TABLE S3 Geographical and physico-chemical data from isolation sites of *Thermus* strains devoid of rubisco

Strain	Isolated from	Rubisco large and small subunit present?	Carbonic anhydrase or Ferripyochelin binding protein[a] present?	Carboxysome proteins (carbon dioxide concentrating mechanism protein)	Temperature (°C) and pH of isolation point	Prevalent geochemical trait of hot spring / thermal area	References
T. thermophilus HB8	Hot spring (Pool B) at Mine, Shizuoka Prefecture, Japan	No	No	No	T = 80<br>pH = 6.3	Very low concentrations of salts and sulfur. Considered as fresh water	(16)
T. thermophilus JL-18	Sandy's Spring West (SSW-18b), Great Boiling Springs geothermal field, Nevada, USA	No	No	No	T = 86.6<br>pH = 7.21	Na^+ = 61.89 mg/L<br>K^+ = 2.69 mg/L<br>Ca^{2+} = 1.95 mg/L<br>Cl^- = 56.60 mg/L<br>SO_4^{2-} = 3.72 mg/L<br>CO_3^{2-} = 0.84 mg/L	(17)<br>(18)
T. scotoductus SA-01	Water sample taken 3.2 km below the surface, Western Deep Levels Ltd. No.1, Carletonville, South Africa	No	No	No	T = 60<br>pH = 8.9	Primarily Na-Cl type water.<br>Na^+ = 204.3 mg/L<br>Ca^{2+} = 47.4 mg/L<br>K^+ = 2.0 mg/L<br>Cl^- = 433.6 mg/L<br>SO_4^{2-} = 10.4 mg/L<br>CO_3^{2-} = 1.5 mg/L	(19)<br>(20)
T. aquaticus Y51MC23	Bath hot spring, Lower Geyser Basin,Yellowstone National Park, USA	No	No	No	T = 88<br>pH = 8.9	Dominant dissolved minerals:<br>Cl^- = 297 mg/L<br>SiO_2 = 244.8 mg/L	Personal communication: Dr. David Mead (Lucigen)
