# Supplementary material for: Whole Genome Comparison of Thermus sp. NMX2.A1 Reveals Principal Carbon Metabolism Differences with Closest Relation Thermus scotoductus SA-01
Source: G3 (Bethesda). 2016 Jul 11;6(9):2791–7. doi: 10.1534/g3.116.032953 (PMC5015936; doi:10.1534/g3.116.032953)
Supplement: Supplemental Material [file supp_g3.116.032953_FileS1.pdf]

Supplemental References.

1. **Chung AP, Rainey FA, Valente M, Nobre MF, da Costa MS.** 2000. *Thermus igniterrae* sp. nov. and *Thermus antranikianii* sp. nov., two new species from Iceland. Int J Syst Evol Microbiol **50:** 209-217.

2. **McGee KA, Sutton AJ, Elias T, Doukas MP, Gerlach TM.** 2006. Puhimau Thermal Area: A Window into the Upper East Rift Zone of Kīlauea Volcano, Hawaii? Pure Appl. Geophys **163:** 837-851.

3. **Allen CC, Albert FG, Chafetz HS, Combie J, Graham CR, Kieft TL, Kivett SJ, McKay DS, Steele A, Taunston AE, Taylor MR, Thomas-Keprta KL, Westall F.** 2000. Microscopic physical biomarkers in carbonate hot springs: Implications in the Search for Life on Mars. Icarus **147:** 49-67.

4. **Hudson JA, Morgan HW, Daniel RM.** 1984. Isolation and characterisation of a new caldoactive filamentous bacterium. FEMS Microbiol Lett **22:**149-153.

5. **Arnórsson S.** 1987. Gas chemistry of the Krísuvík geothermal field, Iceland, with special reference to evaluation of steam condensation in upflow zones. Jökull **37:** 31-47.

6. **Ólafsson M and Bjarnason JO**. 2000. Chemistry of fumaroles and hot springs in the Torfajökull geothermal area, south Iceland, p 1547. Proceedings World Geothermal Congress 2000. Kyushu - Tohoku, Japan.

7. **Bjornsdottir SH, Petursdottir SK, Hreggvidsson GO, Skirnisdottir S, Hjorleifsdottir S, Arnfinnsson J, Kristjansson JK.** 2009. *Thermus islandicus* sp. nov., a mixotrophic sulfur-oxidizing bacterium isolated from the Torfajokull geothermal area. Int J Syst Evol Microbiol **59:** 2962-2966.

8. **Gong N, Chenc C, Xie L, Chena H, Linc X, Zhang, R.** 2005. Characterization of a thermostable alkaline phosphatase from a novel species *Thermus yunnanensis* sp. nov. and investigation of its cobalt activation at high temperature. Biochimica et Biophysica Acta **1750**: 103-111.

9. **Williams RAD, Smith KE, Welch SG, Micallef J.** 1996. *Themus oshimai* sp. nov., isolated from hot springs in Portugal, Iceland, and the Azores, and comment on the concept of a limited geographical distribution of *Themus* species. Int J Syst Bacteriol **46:** 403-408.

10. **Zbanxue S.** 2008. Geothermometry and chemical equilibria of geothermal fluids from Hveragerdi, SW-Iceland, and selected hot springs Jiangxi province, SE-China, 373-402. *In*: Geothermal training programme, Report 1998, 14. Reykjavik, Iceland.

11. **Gestsdóttir K, Geirsson K.** 1990. Chemistry of thermal waters in the Hveragerdi geothermal field. Unpublished Report (in Icelandic). University of Iceland, Reykjavik, 90pp

12. **Anderson JP**. 1978. A geochemical study of the southwest part of the Black Rock Desert and its geothermal areas; Washoe, Pershing, and Humboldt Counties, Nevada. Colo Sch Mines Q **73**:15–22.

13. **Ming H, Yin YR, Li S, Nie GX, Yu TT, Zhou EM, Liu L, Dong L, Li WJ.** 2014. *Thermus caliditerrae* sp. nov., a novel thermophilic species isolated from a geothermal area. Int J Syst Evol Microbiol **64:** 650-656.

14. **Guo Q, Wang Y.** 2012. Geochemistry of hot springs in the Tengchong hydrothermal areas, Southwestern China. J Volcanol Geotherm Res **215-216:** 61-73.

15. **Zhang G, Liu CQ, Liu H, Jin Z, Han G, Li L.** 2008. Geochemistry of the Rehai and Ruidian geothermal waters, Yunnan Province, China. Geothermics **37:** 73–83.

16. **Oshima T, Kazutomo I.** 1974. Description of *Thermus thermophilus* (Yoshida and Oshima) comb. nov., a nonsporulating thermophilic bacterium from a Japanese thermal spa. Int J Syst Bacteriol **24**: 102-112.

17. **Costa KC, Navarro JB, Shock EL, Zhang CL, Soukup D, Hedlund BP**. 2009. Microbiology and geochemistry of Great Boiling and Mud Hot Springs in the United States Great Basin. Extremophiles **13**: 447-459.

18. **Hedlund BP, Mcdonald AI, Lam J, Dodsworth JA, Brown JR, Hungate BA.** 2011. Potential role of *Thermus thermophilus* and *T. oshimai* in high rates of nitrous oxide (N_2O) production in ~80°C hot springs in the US Great Basin. Geobiology **9**: 471-480.

19. **Balkwill DL, Kieft TL, Tsukuda T, Kostandarithes HM, Onstott TC, Macnaughton S, Bownas J, Fredrickson JK.** 2004. Identification of iron-reducing *Thermus* strains as *Thermus scotoductus*. Extermophiles **8**: 37-44.

20. **Onstott TC, Tobin K, Dong H, DeFlaun MF, Fredrickson JK, Bailey T, Brockman FJ, Kieft TL, Peacock, A, White DC, Balkwill D, Phelps TJ, Boone DR.** 1997. Deep gold mines of South Africa: windows into the subsurface biosphere. *In* Instruments, Methods, and Missions for the Investigation of Extraterrestrial Microorganisms. Hoover RB, (ed). Proc. SPIE 3111, San Diego, CA.
